# Galectin-1 as an oncotarget in gliomas and melanomas

**DOI:** 10.18632/oncotarget.408

**Published:** 2011-12-31

**Authors:** Florence Lefranc, Véronique Mathieu, Robert Kiss

**Affiliations:** Laboratoire de Toxicologie, Faculté de Pharmacie, Université Libre de Bruxelles (ULB), Campus de la Plaine, CP205/1, Boulevard du Triomphe, Brussels, Belgium

Galectin-1 is a 14.5-kDa β-galactoside-binding protein that belongs to a 15-member protein family expressed by many different cell types. It exerts major roles in the immune system [1,2] and is involved in the progression of various cancer types [3], including melanomas and gliomas [4]. Galectin-1 displays intracellular (i.e., protein-protein interactions) and extracellular (i.e., protein-oligosaccharide interactions) functions [5]; it displays marked pro-angiogenic [6] and pro-migratory effects in gliomas and melanomas and confers these two tumor types with significant levels of resistance to chemotherapy and radiotherapy [4]. Galectin-1 also aids melanoma [2] and glioma [4] cells in escaping attacks from the immune system, though great hopes have been placed in various types of immunotherapy (including vaccinotherapy) for combating melanomas and gliomas that are associated with dismal prognoses because of their marked intrinsic resistance to proapoptotic stimuli, such as conventional chemotherapy and radiotherapy [4].

With respect to galectin-1-mediated processes of tumor immune escape, it has been demonstrated that galectin-1 secreted by cancer cells kills activated T cells [1] and that targeted disruption of galectin-1 gene expression results in enhanced T cell-mediated tumor rejection [2]. In fact, galectin-1 selectively controls Th1- and Th17-mediated effector functions; it also induces the differentiation of tolerogenic dendritic cells through IL-27- and IL-10-dependent pathways [7].

With respect to galectin-1-mediated pro-angiogenic effects, it has been shown that cancer cells present in hypoxic areas overexpress galectin-1 [4] and that this overexpression leads to marked pro-angiogenic effects [6]. These galectin-1-mediated pro-angiogenic effects are controlled by the ORP150 protein in the cases of glioma [8] and melanoma (unpublished data). Indeed, transiently decreasing galectin-1 expression in glioma cells impairs VEGF maturation (and therefore VEGF secretion from glioma cells) through marked decreases in ORP150 expression [8]. Thus, combining the standard surgery-radiotherapy-temozolomide therapy [4] with anti-galectin-1 and anti-VEGF approaches could lead to significant therapeutic benefits when combating malignant gliomas and potentially metastatic melanomas [4]. Moreover, by itself, galectin-1 promotes angiogenesis [6]. Cancer cells, but not endothelial cells, secrete galectin-1 to exert their hypoxia-mediated pro-angiogenic effects [9]. The uptake of galectin-1 by cultured endothelial cells specifically promotes H-Ras signaling to the Raf/mitogen-activated protein kinase/extracellular signal-regulated kinase (Erk) kinase (Mek)/Erk cascade and stimulates endothelial cell proliferation and migration [9].

Hypoxia also induces increases in resistance to conventional radiotherapy and chemotherapy. Transiently decreasing the expression of galectin-1 in experimental models of melanomas and gliomas significantly increases the therapeutic benefits contributed by the alkylating agent temozolomide, which induces sustained pro-autophagic effects followed by late apoptosis in melanoma and glioma cells [4]. Galectin-1-induced increases in chemotherapy resistance involve distinct biochemical pathways. In melanoma cells, decreases in galectin-1 expression induce heat shock protein 70-mediated lysosomal membrane permeabilization, a process associated with the release of cathepsin B into the cytosol; this is believed to sensitize the cells to the pro-autophagic effects of temozolomide when grafted *in vivo* [4]. In glioma cells, galectin-1 has been found to modulate p53 transcriptional activity and to decrease p53-targeted gene expression, including DDIT3/GADD153/ CHOP, DUSP5 ATF3 and GADD45A [4]. The decrease in galectin-1 expression also impairs the expression levels of other genes implicated in chemoresistance, such as ORP150, HERP, GRP78/BiP, TRA1, BNIP3L, GADD45B and CYR61.

Altogether, these findings clearly point to the fact that decreasing galectin-1 expression (e.g., using monoclonal antibodies or siRNAs [8]) in melanomas and gliomas may weaken the defenses of these two types of cancers against radiotherapy, chemotherapy and immunotherapy/vaccine therapy; may reinforce antiangiogenic therapies; and may weaken both the invasive capacities of these cancers towards neighboring tissues (such as the brain parenchyma for gliomas) and (in the case of melanomas) their metastatic rates [4].

## References

[1] Perillo NL, Pace KE, Seilhamer JJ (1995). Nature.

[2] Rubinstein N, Alvarez M, Zwimer NW (2004). Cancer Cell.

[3] Liu FT, Rabinovich GA (2005). Nat Rev Cancer.

[4] Lefranc F, Mathieu V, Kiss R (2011). World J Biol Chem.

[5] Camby I, Le Mercier M (2006). Glycobiology.

[6] Thijssen VL, Postel R, Brandwijk RJ (2006). Proc Natl Acad Sci USA.

[7] Ilarregui JM, Rabinovich GA (2010). Neuroimmunomodulation.

[8] Le Mercier M, Mathieu V, Haibe-Kains B (2008). J Neuropathol Exp Neurol.

[9] Thijssen VL, Barkan B, Shoji H (2010). Cancer Res.

